# The State of “*Omics*” Research for Farmed Penaeids: Advances in Research and Impediments to Industry Utilization

**DOI:** 10.3389/fgene.2018.00282

**Published:** 2018-08-03

**Authors:** Jarrod L. Guppy, David B. Jones, Dean R. Jerry, Nicholas M. Wade, Herman W. Raadsma, Roger Huerlimann, Kyall R. Zenger

**Affiliations:** ^1^Australian Research Council Industrial Transformation Research Hub for Advanced Prawn Breeding, James Cook University, Townsville, QLD, Australia; ^2^College of Science and Engineering and Centre for Sustainable Tropical Fisheries and Aquaculture, James Cook University, Townsville, QLD, Australia; ^3^Aquaculture Program, CSIRO Agriculture & Food, Queensland Bioscience Precinct, St Lucia, QLD, Australia; ^4^Faculty of Science, Sydney School of Veterinary Science, The University of Sydney, Camden, NSW, Australia

**Keywords:** penaeid shrimps, omics, advanced breeding, aquaculture, genome assembly, linkage mapping, functional genomics, molecular markers

## Abstract

Elucidating the underlying genetic drivers of production traits in agricultural and aquaculture species is critical to efforts to maximize farming efficiency. “*Omics*” based methods (i.e., transcriptomics, genomics, proteomics, and metabolomics) are increasingly being applied to gain unprecedented insight into the biology of many aquaculture species. While the culture of penaeid shrimp has increased markedly, the industry continues to be impeded in many regards by disease, reproductive dysfunction, and a poor understanding of production traits. Extensive effort has been, and continues to be, applied to develop critical genomic resources for many commercially important penaeids. However, the industry application of these genomic resources, and the translation of the knowledge derived from “*omics”* studies has not yet been completely realized. Integration between the multiple “*omics”* resources now available (i.e., genome assemblies, transcriptomes, linkage maps, optical maps, and proteomes) will prove critical to unlocking the full utility of these otherwise independently developed and isolated resources. Furthermore, emerging “*omics”* based techniques are now available to address longstanding issues with completing keystone genome assemblies (e.g., through long-read sequencing), and can provide cost-effective industrial scale genotyping tools (e.g., through low density SNP chips and genotype-by-sequencing) to undertake advanced selective breeding programs (i.e., genomic selection) and powerful genome-wide association studies. In particular, this review highlights the status, utility and suggested path forward for continued development, and improved use of “*omics*” resources in penaeid aquaculture.

## Background

The application of “*omics*” based technologies has provided unprecedented insight into the genetic and functional biology of livestock and crop species, and when integrated within selective breeding programs, “*omics*” techniques have facilitated significant and continued improvements in productivity (Dekkers, 2012; Pérez-de-Castro et al., 2012). In particular, “*omics*” approaches, including genomics, transcriptomics, proteomics, and metabolomics, have been applied widely to elucidate the molecular basis of performance traits (e.g., growth) and overcome poorly understood biological impediments that prevent efficient production (e.g., disease, reproductive failure, and undesired carcass composition) (Rothschild and Plastow, 2008; Taylor et al., 2016). Harnessing genomic information in Holstein Friesians (*Bos taurus*), for example, has led to a doubling of the annual rate of improvement in milk production (García-Ruiz et al., 2016; Taylor et al., 2016), while in layer (egg producing) chickens (*Gallus gallus domesticus*), 16 production traits (i.e., egg size, egg color, and age of sexual maturity) have all seen significant improvements through the application of genomic breeding methodologies (Wolc et al., 2015; Meuwissen et al., 2016). Numerous genetic tests, built upon genomic and transcriptomic research, are now also available for use for commercial applications (e.g., average daily gain, intramuscular fat marbling, meat tenderness, congenital defect screening) in livestock species (Dekkers, 2004). Through these “*omics*” based tests it has been possible for many industries to vastly improve the identification and selection of superior individuals for breeding programs (Dekkers, 2012; Pérez-de-Castro et al., 2012; Taylor et al., 2016).

The transformational effects that “*omics*” research has had on the production of plant and livestock industries is widely acknowledged (see reviews Agrawl and Narayan, 2015; Van Emon, 2015; Taylor et al., 2016). As a result, similar efforts to utilize “*omics*” techniques to improve farmed aquatic species is anticipated to achieve significant improvements to aquaculture production as well (Gjedrem and Rye, 2016). While most terrestrial food production species have already undergone decades of traditional selection, most aquaculture species have only recently been domesticated, with few having been improved through targeted selection programs. In stark contrast to livestock industries, less than 10% of aquaculture production is derived from improved lines (Gjedrem et al., 2012). While the relatively young nature of aquaculture and the scale of the industry has constrained “*omics*” resource development, this also means the latent natural variation of many species and the genetic potential for increased productivity remains unharnessed (Mackay et al., 2009).

Of the aquatic taxa cultured for food worldwide, crustaceans are the fourth most produced aquaculture commodity by weight per year (6.9 million tons), behind finfish (49.8 million tons), marine plants (27.3 million tons), and bivalves (19 million tons) (FAO, 2016). Yet by economic value, crustaceans are the second highest farmed seafood commodity with an annual production value exceeding US$ 35 billion (i.e., ~$5,072 per ton), only surpassed by finfish with an annual economic value of US$ 60 billion (i.e., ~$1,205 per ton) (FAO, 2016). While crustaceans are diverse in species number, penaeid marine shrimp (Family; *Penaeidae*) are the predominately cultured group, with an annual production exceeding 4.8 million tons (FAO, 2017). Of the species farmed, Pacific white-leg shrimp (*Litopenaeus vannamei*) and black tiger shrimp (*Penaeus monodon*) are the primary species cultured, accounting for 80% (~3.9 million tons) and 15% (~0.7 million tons) of total penaeid shrimp production, respectively. The remaining 5% (~0.25 million tons) of production are cumulatively provided by kuruma shrimp (*Marsupenaeus japonicus*), Chinese shrimp (*Fenneropenaeus chinensis*), banana shrimp (*Fenneropenaeus merguiensis*), Indian shrimp (*Fenneropenaeus indicus*), and blue shrimp (*Litopenaeus stylirostris*).

The global penaeid aquaculture industry has demonstrated remarkable growth, increasing in production from a meager 71 tons in the early 1980's, to 4.8 million tons in 2015 (FAO, 2017). Despite the industry's impressive growth, it has faced substantial challenges that still often constrain production. Decades of unsustainable reliance on wild sourced broodstock (for *P. monodon* in particular) (Benzie, 2009), a poor understanding of the biological and genetic basis of production traits (Dunham, 2011), a limited ability to maintain pedigree traceability and a number of devastating global disease outbreaks (Benzie, 2009; Lio-Po and Leaño, 2016), have all impeded industry development. Nevertheless, penaeid shrimp aquaculture is perfectly placed to take advantage of the rapidly emerging suite of “*omics*” techniques that now provide the capacity to explore and address many of the key impediments faced by the industry (Liu, 2007; Gjedrem and Rye, 2016). As such, in a similar manner seen for agriculture, the field of penaeid “*omics*” is now rapidly evolving, fuelled by industry demand for high quality stock, decreased disease risk and improved productivity (Debnath et al., 2016). Furthermore, improvements in breeding techniques and a swell in industry critical mass through increasing centralization of breeding programs (Benzie, 2009) have coalesced to drive the development of critical “*omics*” resources for each of the commercially important penaeid shrimp species (Abdelrahman et al., 2017).

This review focuses on the “*omics*” tools, in particular genomic, transcriptomic, and proteomic resources, that have been developed for penaeids. Specifically, this includes a review of the state of genome sequencing and mapping (linkage, physical, and optical mapping), genomic marker development, functional genomics (transcriptomics and proteomics), and trait mapping studies for penaeid shrimp. This review also provides rationale for the implementation of emerging research methods (e.g., long-read sequencing, high-throughput optical mapping and genotype-by-sequencing), along with suggested approaches to improve the accessibility and application of “*omics*” tools within the global penaeid aquaculture industry.

## Sequencing, assembling, and mapping complex genomes

### Whole-genome sequencing

Comprehensive and complete genomic assemblies are often regarded as the keystone “*omics*” resource for a species due to the significant nucleotide position information they convey, and the ability to leverage genomic data throughout a large variety of further applications. For example, genome assemblies provide a reference base for functional transcriptomic studies, can aid in the positioning of genetic markers, and allow for the examination and characterization of genomic regions of commercial or biological interest. While genomic assemblies are available for a number of aquaculture species [e.g., *Oncorhynchus mykiss* (Berthelot et al., 2014); *Oreochromis niloticus* (Conte et al., 2017); *Lates calcarifer* (Vij et al., 2016); *Ictalurus punctatus* (Liu et al., 2016); *Salmo salar* (Lien et al., 2016)], the comparatively large size (~1.8 Gbp *L. vannamei*, ~2.2 Gbp *P. monodon*) and the highly repetitive nature of penaeid genomes have presented a significant challenge to their assembly (Huang et al., 2011; Baranski et al., 2014; Yu et al., 2015). Likewise, the assembly of penaeid genomes has been further impeded by their large chromosome number (*n* = 44; Table 1) and higher levels of genomic heterozygosity (Abdelrahman et al., 2017) than genomes assemblies derived from inbred domesticated lines of livestock species such as *G. gallus* (International Chicken Genome Sequencing, 2004) or model organisms [e.g., *Mus musculus* (Mouse Genome Sequencing, 2002)]. Despite a number of long standing efforts by numerous research groups (Abdelrahman et al., 2017), no comprehensive genome assembly is available for a penaeid shrimp.

**Table 1:** Summary of the genome size and chromosome number of commercially important penaeids and other major aquaculture species, along with the status of current genome assemblies.

**Species**	**Global production in 2015 (tons) (FAO, 2017)**	**Haploid Chromosome number**	**Genome size (Gb)**	**Sequencing used**	**Assembly size (Gb)**	**Number of scaffold (S) or Contigs (C)**	**Longest scaffold/Contig (Mb)**	**Scaffold N50/ Contig N50 (Mb)**	**References**
*Litopenaeus vannamei*	3,879,786	44	1.78	828 GB Illumina PE^*^ >70GB PacBio	2.6	6007 S	Unknown	0.66	Yu et al., 2015; Xiang, 2016
*Penaeus monodon*	713,318	44	2.17	>140 GB Illumina PE^*^	1.5–3.7	1,168,065–3,064,940 C	0.0021–0.0045	0.0009–0.0019	Baranski et al., 2014; Montenegro et al., 2018
*Marsupenaeus japonicus*	47,726	44	2.4	79.75 GB Illumina PE^*^	1.36	3,244,404 S	0.0015	0.0013	Lu et al., 2017
*Fenneropenaeus chinensis*	44,812	44	2.1	530 GB Illumina PE^*^ >70 GB PacBio	Unknown	Unknown	Unknown	0.15	Kong and Gao, 2005; Xiang, 2016
*Oncorhynchus mykiss*	2,381,576	30	1.9 - 2.18	45 GB Roche 454 128 GB Illumina PE+SE^	1.9	2,018 S	5.4	0.384	Berthelot et al., 2014
*Oreochromis niloticus*	3,930,579	23	1.08	>44 GB PacBio	0.927	2,566 S	37	3.11	Conte et al., 2017
*Lates calcarifer*	76,498	24	0.587	53.6 GB Illumina PE >60 GB PacBio	0.670	24 S	30.7	25.85	Vij et al., 2016
*Ictalurus punctatus*	411,790	29	1.0	88 GB Illumina PE 5.4 GB PacBio	0.783	9,974 S	22.6	7.73	Liu et al., 2016
*Salmo salar*	761,766	29	2.97	13 GB Sanger 326 GB Illumina PE 267 GB Illumina MP^•^ 57 GB PacBio	3.06	9,447 S	>0.038	2.97	Lien et al., 2016

*N.B. No genome assembly information is currently available for Fenneropenaeus merguiensis, Fenneropenaeus indicus or Litopenaeus stylirostris*.

* *PE, Paired End Reads*;

*∧ SE, Single end*;

• *MP, mate pair*.

While genome sequencing efforts have seen dramatic improvement through the development of high-throughput sequencing, the task of resolving, and assembling the many repetitive regions within the penaeid genome (~80%; Abdelrahman et al., 2017) remains particularly challenging. Short read second-generation sequencing methods (i.e., Illumina HiSeq) involve the fragmentation of the genome into millions of 50–300 bp fragments, through which most genome positional based information is lost prior to sequencing. For non-complex genomes, these short sequences can be reconstructed iteratively into near complete chromosomal sections (contigs or scaffolds) by identifying and overlaying sequences that partially overlap each other. However, when short read sequencing methods are applied to highly repetitive regions within the genome, 50–300 bp fragments are of insufficient length to span the repeat regions, resulting in a break down in the iterative tiling approach. This difficultly in building contiguous sequences is exacerbated by diploid genomes with high levels of heterozygosity [e.g., *Ciona savignyi* (Small et al., 2007)] as each multiple assembly paths (each representing one chromosomal copy) cannot be resolved and are either reported as two unique sequences or erroneously joined adjacently to each other (Henson et al., 2012; Pryszcz and Gabaldón, 2016). This effect is evident in the previous short-read assembly by Yu et al. (2015) for *L. vannamei*, in which a *de-novo* assembly of 80–100 bp fragments (at ~41x coverage) resulted in highly fragmented assembly with 4,336,336 scaffolds and maximum scaffold size of only 38,588 bp (Table 1; Yu et al., 2015). Likewise, early *P. monodon* assemblies of 250 bp fragments (~68x coverage) have resulted in 1,168,065–3,064,940 contigs (averaging 1,510 bp in length), and with a maximum contig size of 21,136–45,736 bp depending on the bioinformatics approach used for assembly (Table 1; Montenegro et al., 2018).

Recently, a number of genome assemblies, including those of the Peruvian scallop (*Argopecten purpurtatus*; Li et al., 2018) and for the agricultural crop *Capsicum annuum* (bell-peppers; Hulse-Kemp et al., 2018) have included sequencing data from the novel 10X Chromium platform (10X Genomics) in an effort to decrease ambiguities during genome reconstruction. The preparation of 10X Chromium libraries involves individual barcoding of high-molecular weight DNA fragments (up to 1 million unique barcodes), prior to traditional short-read sequencing. Finally, the 10X Genomics assembler, Supernova, generates an initial global De Brujin graph before utilizing the individual barcoding information to confirm that reads forming each contig are physically linked, and where possible, barcoding information is used to resolve uncertainties (branches, bubbles, and gaps) along each assembled contig (Weisenfeld et al., 2017). However, as Supernova in the first instance still relies upon an iterative sequence-read overlapping approach, difficulties still persistent in assembly of non-model species, or those with complex genomes. For example, early observations from the generation of a 10X Chromium assembly for *P. monodon* has seen Supernova repeatedly fail to reach completion (Montenegro et al., 2018), and suggests this approach may not be successful in resolving the extremely high repetitiveness of penaeid genomes. However, future improved versions of assembly algorithms may allow increase utility of this method for complex non-model species. Likewise, while the additional genome sequencing approach, chromosome conformation capture (Hi-C or ChIP-seq), has been used to improve genome assemblies for goat (*Capra aegarus hircus*) (Bickhart et al., 2017) and maize (*Zea mays*) (Jiao et al., 2017), but requires access to a draft genome which already has a relatively large average size (100kb; Oddes et al., 2018) for effective short read mapping and further improvement of genome scaffolding (Forcato et al., 2017).

As with many species, the traditional genome assembly methods that were initially employed for penaeids have required subsequent amendment to overcome the current difficulties of producing a comprehensive genome assembly. In particular, third-generation sequencing technologies (i.e., Pacific Biosciences Sequel, Nanopore MinION) which generate much longer sequencing reads (mean length over 10 kbp), are increasingly being utilized to improve the accuracy and completeness of genome assemblies in many plant and livestock species [e.g., pineapple, *Ananas comosus* (Ming et al., 2015); rice, *Oryza sativa* (Brozynska et al., 2016); and goat, *C. a. hircus* (Bickhart et al., 2017)]. Even more so, recent improvements in the sequencing chemistry, rate of throughput and accuracy of base calling have allowed the more cost effective generation of large volumes of long read data (e.g., 30–50x coverage, with an average ~10 kb read length; Phillippy, 2017). As such, aquaculture species are now looking toward long read sequencing technologies (LRSTs) to resolve the assembly of repetitive and complex genomes (i.e., Nile tilapia, barramundi, channel catfish, Atlantic salmon) (Liu et al., 2016; Vij et al., 2016; Conte et al., 2017). LRSTs have most notably helped to achieve a near chromosomal-scale assembly of the large Atlantic salmon genome, which contains complex remnants of three separate whole-genome duplication events (Lien et al., 2016).

Recently published details of the ongoing effort to complete the *L. vannamei* have noted the contig N50 (minimum contig length to cover 50% of the genome) has been reported (Abdelrahman et al., 2017) to have increased from 547 to 660,000 bp and the total number of scaffolds decreased from 4,336,336 to 6,007 through the incorporation of Pac Bio sequencing reads (Yu et al., 2015; Abdelrahman et al., 2017). Long read sequencing approaches are currently also being employed in assembly of the *P. monodon* genome with the aim to achieve similar improvements in genome assembly quality as seen in *L. vannamei* (Montenegro et al., 2018). Details have been released on a current effort to assemble the *F. chinensis* genome (Xiang, 2016); however, beyond the scaffold N50 (154.2kb) and sequence data generation (530 GB short read, 70 GB long read), the final assembly quality metrics are currently unavailable to the public. Likewise as both *L. vannamei* and *P. monodon* genomes are yet to be published, the details of their final assembly quality (genome completeness, scaffold size, average genome coverage), given the technology now available, still remain to be seen. It is likely the most successful assemblies will rely upon a “hybrid assembly” approach, utilizing short-read sequencing to correct the high error rate base-calling seen in long read sequencing, prior to assembly of “corrected” long reads (Sedlazeck et al., 2018). In construction of a *P. monodon* genome, difficulties have been encountered with obtaining sufficient high molecular weight DNA from shrimp tissues while avoiding contamination of DNA with polysaccharides that can interfere with long-read sequencing devices (Montenegro et al., 2018), further development of shrimp specific high molecular weight DNA extraction protocols are required.

While genomic-survey sequencing has been conducted in Kuruma shrimp (*M. japonicus*) at low coverage (33.23x short read genome coverage; Lu et al., 2017), genome assemblies for the Kuruma shrimp, or other commercially important penaeid species such as the Indian shrimp (*F. indicus*) are still yet to be undertaken. Additional penaeid assemblies are likely to follow once the *P. monodon, L. vannamei*, or *F. chinensis* genomes are available to be used for reference-guided genome assembly. Release of fully annotated and integrated genome assemblies in publicly accessible formats (e.g., as genome browsers in NCBI) should be a primary focus of the penaeid “*omics*” research community.

### Linkage mapping of genetic markers

Linkage maps are highly versatile genomic resources which provide a wealth of genomic information and facilitate the examination of the underlying genetic architecture of commercially and biologically important traits. As such, the development of linkage maps for penaeids (Table 2) has recently seen significant research effort with a number of maps now available for *P. monodon* (Wilson et al., 2002; Wuthisuthimethavee et al., 2005; Staelens et al., 2008; Baranski et al., 2014), *L. vannamei* (Pérez et al., 2004; Alcivar-Warren et al., 2007; Zhang et al., 2007; Du et al., 2010; Andriantahina et al., 2013; Gonçalves et al., 2014; Yu et al., 2015; Jones et al., 2017a), *M. japonicus* (Li et al., 2003; Lu et al., 2016b), and *F. chinensis* (Li Z. et al., 2006; Sun et al., 2008; Tian et al., 2008; Liu et al., 2010; Wang et al., 2012; Zhang et al., 2013). Linkage map construction involves the genetic analysis of family groups (both parents and progeny), to allow the identification of the recombination pattern of polymorphic markers, and in turn, the calculation of a relative genomic position for each marker. When markers are positioned accurately within discrete linkage groups (with each group indicative of a chromosome), the genomic map provides a robust method to validate and error correct draft genome assemblies (Fierst, 2015), and also provides critical tools for the study of trait and genome architecture (as discussed in section Dissecting and Exploiting the Genetic Variation Underlying Phenotypes).

**Table 2:** Linkage maps available for penaeid shrimp, organized by species and marker used.

Species ^*^	**Marker type**	**Individuals used**	**Families used**	**Linkage groups covered (n/44)**	**Markers mapped**			**Total map length (cM)**			**Average inter-marker distance (cM)**			**Genome coverage**			**Software used**	**Reference**
					**Male**	**Female**	**Sex av**	**Male**	**Female**	**Sex av**	**Male**	**Female**	**Sex av**	**Male**	**Female**	**Sex av**		
*L. vannamei*	SNPs	205 •	1	44	4,201	4,396	6,146	6,143.95	5,657.42	4,271.43	1.46	1.29	0.7 ^**^	97.49%	97.71%	98.39%	HighMap	Yu et al., 2015
*L. vannamei*	SNPs	631∧	49^θ^	44	NA	NA	4,817	4,522.30	4,530.60	4552.50	NA	NA	0.97	NA	NA	98.12%	Carthagene V1.3	Jones et al., 2017a
*L. vannamei*	SNPs	144∧	3	45	413	418	418	2,130	2,071	2,262.3	NA	NA	NA	37.25%	48.27%	NA	Crimap	Du et al., 2010
*L. vannamei*	AFLP	42 •	1	51 ♂, 47♀	182	212	394	2,116	2,771	NA	15.6	17.1	NA	59%	62%	NA	MapManagerQTX MapMaker v 3.0	Pérez et al., 2004
*L. vannamei*	AFLP	192∧	2	18	NA	NA	157	NA	NA	NA	NA	NA	NA	NA	NA	NA	GQMOL	Gonçalves et al., 2014
*L. vannamei*	AFLP, MSat	94 •	1	45	267	319	NA	3,220.9	4,134.4	NA	14.5	15.1	NA	69%	75%	NA	MapMaker v 3.0	Zhang et al., 2007
*L. vannamei*	AFLP, MSat	43∧	1	49	NA	NA	451	NA	NA	313.9	NA	NA	7.6	NA	NA	72.63%	Mapmanager QTXb20	Andriantahina et al., 2013
*P. monodon*	SNPs	1,024 •	7	44	3,486	3,592	3,959	2,917	4,060	NA	0.9	1.2	NA	91%	82%	NA	TMAP	Baranski et al., 2014
*P. monodon*	AFLP	126∧	3	20	NA	NA	116	1,412	NA	NA	22	NA	NA	NA	NA	NA	MapMaker	Wilson et al., 2002
*P. monodon*	AFLP	344 •	3	44♂, 43♀ 21 sex ave.	757	494	70	2378	2362	NA	2.1	2.8	NA	NA	NA	NA	Joinmap 3.0	Staelens et al., 2008
*P. monodon*	MSat, EST, SCAR	76 •	1	9	NA	NA	27	NA	NA	103.6	NA	NA	3.8	NA	NA	NA	Joinmap 2.0	Wuthisuthimethavee et al., 2005
*P. monodon*	MSat, EST	42 •	1	47♂, 36♀	157	111	NA	1101.0	891.4	NA	7.0	8.0	NA	NA	NA	NA	Mapmaker	Maneeruttanarungroj et al., 2006
*M. japonicus*	SNPs	150 •	1	41	3,927	5,849	9,298	3326.19	3,127.23	3,610.90	0.847	0.535	0.388	NA	NA	99.06%	JoinMap 4.0	Lu et al., 2016b
*M. japonicus*	AFLP	102 •	1	43♂, 31♀	217	125	NA	1780	1026	NA	NA	NA	NA	NA	NA	NA	MapMaker v 3.0	Li et al., 2003
*F. chinensis*	SNPs	100 •	1	21	115	119	180	879.7	876.2	899.3	9.4	8.9	5.4	51.94%	53.77%	NA	JoinMap 3.0	Zhang et al., 2013
*F. chinensis*	AFLP	100 •	1	36♂, 35♀	194	197	NA	1,737.3	2,191.1	NA	11.0	13.5	NA	68.1%	69.6%	NA	MapMakerv 3.0	Li Z. et al., 2006
*F. chinensis*	AFLP	110∧	1	35♂, 28 ♀ 44 sex ave.	144	103	216	1617	1090	1772.1	16.36	14.53	10.42	44.41%	47.61%	63.17%	MapMaker v 3.0	Tian et al., 2008
*F. chinensis*	MSat, RAPD	82 •	1	10 ♂, 8 ♀	46	49	NA	1,144.6	1,173	NA	12.05	11.28	NA	62.01%	59.36%	NA	Mapmaker v 3.0	Sun et al., 2008
*F. chinensis*	AFLP, MSat, RAPD	100 •	1	47	NA	NA	354	NA	NA	4,580.5	NA	NA	11.3	NA	NA	75.8%	Joinmap 3.0	Liu et al., 2010

* *L. vannamei (Litopenaeus vannamei), P. monodon (Penaeus monodon), M. japonicus (Marsupenaeus japonicus), F. Chinensis (Fenneropenaeus chinensis)*;

** *2.67 cM when 0 cM inter-marker distances are removed; SNPs, Single Nucleotide Polymorphisms; MSat, Microsatellite or Simple sequence repeats; EST, Expressed Sequence Tag; SCAR, Sequence Characterized Amplified Region; RAPD, Random Amplified Polymorphic DNA; AFLP, Amplified Fragment Length Polymorphism*;

∧ *Phase known*;

• *Phase unknown*;

θ *Half-sib families*;

♂ *male map*;

♀ *female map*.

Early versions of linkage maps for penaeid shrimps contained a limited number of genetic markers (Table 2) due to the inherent difficulties in isolating informative polymorphic markers (as discussed in section Development and Applications of Polymorphic Markers). More recently, however, the linkage maps available for penaeids have improved dramatically, as it has now become possible to generate and simultaneously map the position of thousands of polymorphic SNP markers (Baranski et al., 2014; Yu et al., 2015; Lu et al., 2016b; Jones et al., 2017a). Linkage maps are now available that include between 3,959 and 9,298 markers and cover all 44 chromosomes of the penaeid genome (Baranski et al., 2014; Yu et al., 2015; Lu et al., 2016b; Jones et al., 2017a). When compared to earlier maps (Wilson et al., 2002; Li et al., 2003; Pérez et al., 2004; Wuthisuthimethavee et al., 2005; Li Z. et al., 2006; Maneeruttanarungroj et al., 2006; Alcivar-Warren et al., 2007; Zhang et al., 2007; Staelens et al., 2008; Andriantahina et al., 2013; Gonçalves et al., 2014) that contained 27 to 451 markers, distributed across between 8 and 51 linkage groups, the increase in density of polymorphic markers recently achieved has dramatically increased the applicability of these resources (i.e., assisting genomic assembly, examining the genetic architecture of traits, and undertaking comparative mapping).

As such, the construction of linkage maps has provided a number of interesting insights into the genomic structure of penaeids. For example, Baranski et al. (2014) constructed maps for *P. monodon* in which the female-specific map was substantially shorter than the male-specific map (2,917 vs. 4,059 cM). Alternatively, in *L. vannamei*, Pérez et al. (2004) and Zhang et al. (2007) both obtained longer maps for females than males (4,134 vs. 3,221 cM, and 2,771 vs. 2,116 cM, respectively), indicating that there may be higher recombination in males. In the absence of obvious karyotype size and number differences between male and female shrimp (You et al., 2010), the observed differences in map lengths suggests that sex-biased recombination occurs in penaeids, but may occur in a species dependent manner (Baranski et al., 2014). Further detailed map construction is required to validate and understand this difference in recombination between sexes, along with identification of genomic recombination hotspots. Patterns of recombination, specifically in areas of the genome containing genes of commercial importance, may affect the accuracy of and impact derived from genome-informed selection programs (Meuwissen et al., 2001; Habier et al., 2007; Goddard, 2009). Construction of linkage disequilibrium unit (LDU) or haplotype block maps have been required to obtain an understanding of recombination across each chromosome of the genome of livestock and crop species (Habier et al., 2007; Amaral et al., 2008). However, given previous constraints generating dense linkage maps, no LDU or haplotype information is currently available for penaeid shrimp, but as advanced selection programs begin to be implemented in the industry generating haplotype maps should be a significant focus of future studies. Alternatively, haplotype information has been generated through either long or short-read (10x Genomics or Illumina) whole genome resequencing studies (multiple individuals at 5–10x coverage per individual) in many species including cattle (Daetwyler et al., 2014). However as sequence derived haplotype maps rely upon the availability of error-free genome assemblies, this approach is not currently feasible for penaeids.

To date, comprehensive comparative genomics studies examining genome synteny/divergence, chromosomal evolution, and structuring between penaeid species has been limited. Excluding the *L. vannemai* map published by Yu et al. (2015), integration of linkage maps with the fragmented genome sequence data has yet to be completed. Furthermore, novel marker sets have been developed and utilized for most maps available, with few maps [i.e., Maneeruttanarungroj et al. (2006) building upon Wilson et al. (2002), and Jones et al. (2017a) building upon Du et al. (2010), and Ciobanu et al. (2010)] having integrated previous mapping resources into the updated maps. Jones et al. (2017a) has undertaken early comparisons of genomic synteny between *P. monodon* and *L. vannamei* [along with other *L. vannamei* maps (Du et al., 2010; Yu et al., 2015)], predominately observing conservation of chromosomal organization. However, due to only a small number of common markers (275 SNPs) between both maps (Baranski et al., 2014; Jones et al., 2017a), it was not possible to thoroughly investigate chromosomal rearrangement, gene order, or differences in recombination (e.g., sex biased, genomic hotspots). While a substantial amount of research effort has been applied to date on developing maps (Table 2), further integration between linkage maps would provide an opportunity to rapidly increase map density for each species. A number of previous maps are not easily accessible, despite, in some cases, providing access points at the time of publication (e.g., Wilson et al., 2002; Maneeruttanarungroj et al., 2006). Ensuring long-term access to mapping resources is critical to maximize the use of previous resources and also enable detailed comparative genomic studies to be undertaken.

To extend the utility of linkage maps and provide the ability to understand the fine scale genomic drivers of commercial traits (e.g., genome-wide association, multifactorial association) it is necessary to increase marker density contained in maps for many species. Maps available for *P. monodon* (Baranski et al., 2014) and *L. vannamei* (Yu et al., 2015), have average inter-marker distances between 0.9 and 0.7 cM, respectively, across different map iterations. While this is a significant advance from earlier published maps, 1 cM equates to an estimated physical genome distance of ~400–600 Kb for penaeids [*P. monodon* 395 Kb/cM (Baranski et al., 2014); *L. vannamei;* 598.89 Kb/cM; (Yu et al., 2015), *M. japonicus* 657.89 Kb/cM (Lu et al., 2016b)], and presents a significant challenge when looking to characterize potential genes or genomic regions underlying findings of trait association studies. The linkage map available for *M. japonicus* (Lu et al., 2016b) achieved the highest map density with an average inter-marker distance of 0.39 cM, representing an estimated physical distance between markers of ~230–257 Kb (Yu et al., 2015; Lu et al., 2016b). However it should be noted, only 41 linkage groups were reported in this map, rather than 44 observed in other penaeid species (Table 2).

To increase the density and decrease the interval between markers, a number of strategies can be applied. Further genotyping of families, and individuals in each family will provide additional observations of informative meiotic recombination events or integrate unplaced (i.e., orphaned) markers into existing maps (Fierst, 2015). Current maps have either had relatively few individuals from each family, yet many families included [e.g., 8–33 individuals per family from 49 families (Jones et al., 2017a)], or include many individuals from a smaller number of families [e.g., 100 progeny from one family (Lu et al., 2016b)], with both approaches restricting the number of informative meiotic events available for placement of each marker on the map (Jones et al., 2017a).

Likewise, incorporating additional genotyped individuals will assist in teasing apart the positioning and order of “binned” markers (those co-segregating to a single map location) (Fierst, 2015). In the *L. vannamei* linkage map by Jones et al. (2017a) 4,817 markers were mapped to 1,752 unique locations (bins), with an average of 2.75 markers per location. It is likely maps by Baranski et al. (2014), Yu et al. (2015), and Lu et al. (2016b) have observed the same effect, however, it is uncommon that the proportion of “binned” markers is discussed, with Jones et al. (2017a) being the only publication to provide details of both average (0.97 cM) and “non-zero” average inter-marker distance (2.67 cM). The discrepancy between total mapped markers and unique locations should be clearly reported with all linkage maps, as determining distribution of unique locations is important to integration of genome sequence information (Fierst, 2015), understanding of the power and resolution available to study commercial traits, as well as in reducing bias (from uneven marker distribution) in the calculation of genomic estimates of breeding value (Mathew et al., 2018). Utilizing increasingly cost effective genotyping strategies (e.g., genotype by sequencing; see section Dissecting and Exploiting the Genetic Variation Underlying Phenotypes) will facilitate a large number of individuals/families to be genotyped and aid efforts to achieve fine grain marker placement.

The anchoring of long-read sequences produced through ongoing genome assemblies can be used to tease apart the location of markers placed in unordered bins (Fierst, 2015). However, to integrate linkage maps with genome assemblies, markers are required to be sufficiently close together to place multiple markers on each genomic scaffold. It has been indicated that linkage maps have been integrated within genomic assembly strategies for *L. vannamei* (Yu et al., 2015; Xiang, 2016), however, an integrated resource has yet to be released in an accessible format. Once completed, these integrated mapping resources will provide an intermediate point for comparison between otherwise disparate maps generated through different genotyping assays or with different marker types, and will provide a vital resource for comparative genomic studies and quantitative trait mapping.

### Physical and optical genome mapping

While linkage mapping and LRSTs promise to provide a significant increase in the quality of genome assemblies for penaeids, it is still difficult to produce a chromosome level assembly through the addition of these methods alone (Fierst, 2015; Howe and Wood, 2015). The generation of physical map information provides an additional approach for genome scaffolding alongside linkage maps and LRSTs (Fierst, 2015). Bacterial artificial chromosome (BAC) based physical mapping has been utilized previously in the genome assemblies of *Drosophila* (Myers et al., 2000), domestic cattle (*Bos taurus*) (Zimin et al., 2009), and barramundi (*Lates calcarifer*) (Vij et al., 2016), to aid in anchoring short scaffolds and improve the overall assembly quality and accuracy. BACs require the ligation of long fragments of DNA (200 kbp) into bacterial plasmids, after which colonies could be individually screened. Fragments are then reisolated and sequenced individually by either long or short read sequencing, with the overall aim to reduce the complexity of the sequence reassembly process. However, due to the high cost and difficulty in isolating sufficient unique BAC fragments, there is only two partial BAC-end libraries available for *L. vannamei* (Zhang X. et al., 2010; Zhao et al., 2012), one partial Fosmid library for *P. monodon* (random physical sheared ~40 kb library; Huang et al., 2011) and one small insert BAC library for Kuruma shrimp (Koyama et al., 2010). While Yu et al. (2015) have mapped the available BAC-end library to the *L. vannamei* linkage map, providing a valuable resource for fine trait mapping in QTL studies (See section Quantitative Trait Loci Mapping), it is unclear to what degree this data has aided assembly quality (Yu et al., 2015; Xiang, 2016).

In the study of many other species (Howe and Wood, 2015; Vij et al., 2016; Bickhart et al., 2017), the physical mapping information that BACs provide has instead been replaced by modern methods of fluorescent *in situ* hybridisation (FISH) and optical mapping. Through these methods, gene-specific fluorescent probes are developed to enable the direct visualization of a gene's location on a chromosome. By utilizing a combination of multiple probes it is possible to measure the physical distances between genes, validating gene order and distances established from linkage maps and genome assemblies. For example, physical maps can be utilized to identify and correct regions of whole genome assemblies which have ambiguously assembled regions (i.e., repeat regions, unknown gap lengths, chimeric scaffolds), or have included misassembles (i.e., false joins, inversions, repeat collapsing) (Fierst, 2015; Howe and Wood, 2015). In several cases, due to the length of optical mapping fragments, it is possible to reconstruct reference sequences of entire chromosomes (Howe and Wood, 2015). Yet like BACs, the development of FISH-based optical maps has been limited within aquaculture species, with no optical maps available for penaeids. This is a result of their initial high cost, low throughput, and relatively limited data generation. Currently, only proof of concept studies have been completed with FISH methods on penaeid species, with a single chromosome-specific FISH probe developed for *F. chinensis* (Huan et al., 2010) and four TAACC repeat specific FISH probes developed for *L. vannamei* (Alcivar-Warren et al., 2006).

While the high cost of initial manual optical mapping techniques has prevented their widespread use, optical mapping techniques are being increasingly refined to assist with the assembly of complex and repetitive genomes. The automation of optical mapping technology has led to the development of a number of commercial platforms, such as Irys (BioNano Genomics) and Opgen (Argus) which can deliver high-throughput physical map data at reduced costs (Howe and Wood, 2015; Sharp and Udall, 2016). High-throughput optical mapping provides a physical fingerprint of each DNA molecule, through the fluorescent labeling of multiple restriction sites or “nick sites” along large linear DNA fragments (20 Kbp to 3 Mbp in length; Sharp and Udall, 2016). With the inherent difficulties observed in constructing a complete chromosomal-scale genome assembly for penaeids, integrating physical mapping information into the assembly process will prove critical to resolving repetitive regions and producing a successful final draft assembly. Given the diverse range of genomic resources (i.e., linkage maps, optical mapping, short, and long range sequencing) now available to address complex assemblies, a completed peneaid genome assembly is likely to be achieved quite soon.

## Development and applications of polymorphic markers

### The replacement of traditional genomic markers

A large amount of research effort over the past three decades has been dedicated to developing a diverse range of traditional genomic markers (i.e., allozymes, microsatellites, AFLP, RFLP markers) for many penaeid species. Their development has increasingly provided the tools to assess wild source populations, manage family lines, and undertake cursory broad-brush assessment of the heritability and genetic architecture of traits. However, many of these markers exhibit caveats which have been reviewed extensively by Benzie (1998, 2009) and Wang et al. (2004). Ultimately, due to high development costs for each marker, the increasing complexity of breeding programs (Jerry et al., 2004; Sellars et al., 2014) and an inability to tease apart complex polygenic production traits, these markers have rapidly fallen out of favor in penaeid research programs and have seen a reduced application within industry. Instead the traditional marker sets described above, are being replaced by powerful and low cost modern marker panels, overcoming the many shortfalls of traditional marker sets.

### Modern genomic markers and solid state arrays for breeding programs

Single nucleotide polymorphisms (SNPs) are the most abundant polymorphic markers contained within genomes and are fundamental to modern genomic studies. Large genome sequencing and resequencing projects have identified over 9 million SNPs in the cattle (*B. taurus*) genome (Xu Y. et al., 2017) and 7 million SNPs in chickens (*G. gallus*) (Rubin et al., 2010). Within aquaculture species, over 9.7 million SNPs were identified in Atlantic salmon (*Salmo salar*) through whole genome sequencing of 20 individuals (Yáñez et al., 2016). Similarly in the channel catfish (*Ictalurus punctatus*) and barramundi (*Lates calcarifer*), 8.6 and 5.6 million SNPs were identified through genome re-sequencing of 1,213 and 61 individuals, respectively (Vij et al., 2016; Zeng et al., 2017). In addition to SNP discovery, commercial genotyping arrays containing 50,000 to >500,000 markers have been developed as tools for the study and management of many primary production species, including cattle [BovineSNP50 assay (Matukumalli et al., 2009)], sheep [*Ovis aries*; OvineSNP50 Bead Chip (McEwan and Consortium, 2009); OvineSNP600 Bead Chip (Anderson, 2014)], and wheat [*Triticum* spp.; Axiom HD (820K) wheat genotyping array (Winfield et al., 2015)], as well as aquaculture species, including catfish [250K array (Liu et al., 2014), 690K array (Zeng et al., 2017)] and Atlantic salmon [~130K array (Houston et al., 2014)]. While a number of genome sequencing programs are underway for penaeids species (Yu et al., 2014, 2015; Xiang, 2016), they have yet to develop genotyping arrays of greater than 10,000 markers.

The development and use of high density genotyping arrays for penaeids has been impeded by a number of factors. Most critically, the fundamental genomic resources (high quality genomic assemblies and genomic re-sequencing data) that underpin the development of these high density arrays are not currently as refined as they need to be for penaeid species. With the exception of recent work by Yu et al. (2015), and Lu et al. (2016b) which utilized genotype-by-sequencing approaches (section Genotype by Sequencing as a Low Cost Approach) to produce 114,829 and 28,981 SNPs, studies have largely aimed to generate SNP markers through whole transcriptome studies (Baranski et al., 2014; Yu et al., 2014; Jones et al., 2017a). While this is an effective approach for marker discovery, it also requires costly additional genotyping of samples across the discovered SNPs to validate their performance. To date, only two studies have produced validated SNP genotyping arrays; Baranski et al. (Baranski et al., 2014) for *P. monodon*, and Jones et al. (Jones et al., 2017a) for *L. vannamei* (now sold commercially as the Infinium ShrimpLD-24 v1.0 Bead Chip). These arrays present a significant advancement for these two penaeid industries, containing 6,000 and 6,400 SNPs respectively. As both arrays are based on type-1 SNPs (i.e., genic, rather than intergenic), many of the SNPs have been annotated with putative genes (62 and 47% respectively) providing a significant resource for future studies. Likewise, both studies have also confirmed the distribution of markers through linkage mapping, providing the foundations for further trait mapping studies (Robinson et al., 2014; Khatkar et al., 2017b). Markers selected for both arrays represent only a small fraction of the total markers identified (Baranski et al., 2014; Jones et al., 2017a), with 473,600 and 234,452 SNP identified, respectively. This represents a significant pool of markers that could be utilized in ongoing studies to increase array density if required.

While it is evident that the modern high density genotyping arrays are powerful tools, and their development marks a step forward for penaeid aquaculture, the cost of genotyping (~AU$ 40-100 per individual) on the platforms selected (Illumina I-Select and Bead chip arrays) remains generally too high for large-scale routine use by the penaeid aquaculture industry. Instead, for species where these high density arrays have been successfully implemented (most notably in agriculture and Atlantic salmon), the price of genotyping has been reduced by increased demand from centralized, cooperative, or national breeding programs which have the advanced technical expertise to utilize the data obtained. The price impediment has been further circumvented by removing the farm by farm requirement for genotyping high numbers of broodstock in each subsequent generation (Thodesen and Gjedrem, 2006; Janssen et al., 2018). In many cases these shrimp breeding programs, are operated by private enterprises that have a either specialized focus on production of seedstock (centralized nucleus) or operate a fully integrated production model (seedstock production through to harvest), however, some government or collaboratively funded programs do exist (Benzie, 2009). In private enterprise operations, the utilization of genotyping resources is reliant upon obtaining an increase in the achievable sale price of seedstock through achieving improved selection practices, as seedstock are marketed upon predicted improved grow-out performance (Janssen et al., 2017). Nevertheless, cost of genotyping has to be outweighed by increased economic returns achieved. Without centralized or highly concentrated breeding in place for all species and countries, and until a point when genotyping arrays can be delivered at a more industry accessible price, the use of these genotyping resources will remain confined to a research setting (Robledo et al., 2017). A number of sophisticated and large scale breeding programs are now underway for *L. vannamei* and *P. monodon* and hold the most promise for high density solid-state genotyping resources to be fully utilized (Castillo-Juárez et al., 2015; Li et al., 2016; Campos-Montes et al., 2017). Early DNA parentage arrays for penaeid shrimp were largely based on microsatellite DNA markers (Alcivar-Warren et al., 2003; Jerry et al., 2004, 2006), however, with the advent of next-generation sequencing it has been possible to more easily isolate SNPs. Consequently, parentage testing SNP arrays have now been developed for *P. monodon* as an intermediate step to industry application of cost-prohibitive high-density SNP genotyping (Sellars et al., 2014). While these ultra-low density arrays, containing 63 and 59 SNPs each, or 122 SNPs as a combined array (Sellars et al., 2014), lack the number of markers required for advanced genomic applications (e.g., genomic selection, GWAS, QTL mapping, and calculating genomic relationship matrixes), they do provide increased power and utility over traditional genomic markers for pedigree assignment (see section Applying Markers for Breeding Population Management) (Henshall et al., 2014). The development of a similar range of SNP arrays is currently feasible for all other commercially important species as extensive transcriptome data has been made publically available for *M. japonicus, F. chinensis, F. merguiensis, F. indicus*, and *L. stylirostris*. Furthermore, for these species which lack any modern genotyping tools, replicating a low density array could provide a cost effective approach for guided farm management and improvement of shrimp production (Vandeputte and Haffray, 2014). Ultimately in the selection of a genotyping method for commercial applications the time required for sample processing, genotyping, and data analysis needs to be considered, particularly given the window between pre-selection of candidate broodstock at harvest, and final breeding selection and spawning is short (often less than 3–6 months). Genotyping technologies such as the Illumnia Bead Chip or Sequenom I-plex systems that can achieve both high-throughput and short turnaround times are currently most conducive to rapid decision making required in shrimp breeding.

### Genotype by sequencing as a low cost approach

The regular use of solid state genotyping arrays across thousands of individuals within commercial settings is currently out of reach for the majority of the penaeid aquaculture industry. Nevertheless, the advent of a number of novel genotype-by-sequencing (GBS) approaches (e.g., double digest restriction site associated DNA, ddRAD; Diversity Arrays Technology Sequencing, DArT-Seq; specific-locus amplified fragment sequencing, SLAF) holds promise to rapidly provide a genotyping platform for less than AU$ 40 per individual. The particular advantage of GBS approaches is the ability to discover and genotype markers (“*de-novo* marker discovery”), without requiring reference to existing genomic information (genomic sequence, transcriptomes). A number of GBS approaches (SLAF-tag, RAD, and DART-Seq) have been utilized in the study of penaeid species, with 25,140 and 23,049 markers obtained separately for *L. vannamei* (Yu et al., 2015; Wang et al., 2017), and 28,981 markers obtained for *M. japonicus* (Lu et al., 2016b). While these markers have been applied to generate linkage maps (Yu et al., 2015; Lu et al., 2016b), undertake QTL mapping (Yu et al., 2015; Lu et al., 2016b), and estimate genomic prediction accuracy (Wang et al., 2017), they have yet to be further utilized within the industry for routine genotyping applications. Further refinement of GBS protocols along with integration of targeted fragment capture technology [e.g., Bates Probes (Ali et al., 2016), Molecular Inversion Probes (Niedzicka et al., 2016)] can deliver arrays with 1,000–10,000 markers for under AU$ 20 per individual.

The first GBS-based targeted fragment capture technology SNP assay (containing ~5,000 markers) for a penaeid species is in final stages of development (Guppy et al., 2018), and aims to provide the *P. monodon* industry the first cost-effective opportunity to undertake genomic selection. Furthermore, as with solid state sequencing platforms, GBS-based assays can be used to undertake genomic relationship calculations and examine the genome structure and architecture of traits (Zenger et al., 2017). With the development and use of GBS arrays becoming more frequent in aquaculture as a low cost alternative to solid state arrays (Holman et al., 2017; Robledo et al., 2017), it is likely that similar marker panels with tailored density and composition of markers will be developed for other penaeid species (Wang et al., 2017).

The utility of GBS for aquaculture species is reviewed in detail by Robledo et al. (2017), and for genomics more broadly in Andrews et al. (2016). While there are many benefits of GBS based genotyping in terms of flexible design, and improved accessibility through lower cost, it must be noted that, when compared to solid state arrays (Illumina I-select and bead chip arrays or Affymetrix Axiom Arrays), the genotype data obtained can be of lower quality (e.g., null alleles, read-depth dependant genotype accuracy, high missing data) if not filtered through appropriate quality control measures (Andrews et al., 2016). To account for this, custom pipelines [e.g., STACKS (Catchen et al., 2013), TASSEL (Glaubitz et al., 2014), DARTR (Bernd et al., 2018)] are being continuously refined and will provide valuable software resources to more effectively utilize data generated by this genotyping method. Likewise, as the generation of genotyping assays through GBS approaches is still an emerging area of research, the process of providing functional and validated assays is currently more complex than solid-state arrays.

### Applying markers for breeding population management

Managing a sufficiently large and diverse core population of breeding individuals has always been a significant challenge for the shrimp industry and there has not been a cost-effective method to recover pedigree data on-farm. Programs relying on the separate rearing of family lines have been overshadowed by high infrastructure costs and an inability to hold sufficient numbers from each family line to make effective selection decisions. In addition, by raising individuals in separate rearing tanks, or ponds, farmers inadvertently introduce confounding environmental effects to any comparative estimates of performance between rearing systems (Sonesson and Ødegård, 2016). Furthermore, it is not possible to maintain genealogical traceability with external tags as shrimp molt as they grow, and are too small in size (~2 mm) prior to stocking into commercial ponds for feasible use of internal tags (visible implant elastomer or passive integrated transponders). Overlooking accurate tracking of pedigree has on a number of occasions led to poor management strategies being employed, and inbreeding being observed within penaeid farms (Garcia et al., 1994; Wolfus et al., 1997; Xu et al., 2001; Moss et al., 2007; Dixon et al., 2008; Knibb et al., 2014).

Nevertheless, as access to genomic markers has increased, breeding programs have been provided with the tools (i.e., genetic pedigree assignment and genetic diversity assessment) to assist in farm management. While these resources have improved substantially, industry use for penaeids has been inconsistent. The cost of genotyping (high-density solid state arrays), lack of genotyping power (microsatellites), or a combination of both factors, have been the primary impediments to wider spread use of many existing marker sets (Vandeputte and Haffray, 2014). Importantly, however, both low-density solid state arrays and GBS assays provide the penaeid industry with a cost-accessible and powerful set of tools for real world application. For instance, as demonstrated by Henshall et al. (2012, 2014), proportions of communally reared families can be determined from pooled samples (i.e., DNA pooling of multiple individuals) that have been genotyped with low density SNP arrays (Sellars et al., 2014). Both low-cost genotyping and DNA pooling form an important progression for the industry as they allow phenotypic data from commercial ponds to be linked back to broodstock families without the need to physically track pedigree or undertake isolated rearing of individual families. By doing so, the confounding effects of variable rearing conditions upon estimates of performance can be reduced (Kinghorn et al., 2010). When phenotypic information is combined with individual genetic pedigree assignment of progeny, superior broodstock can be selected to produce improved subsequent generations (Henshall et al., 2014), as well as estimate genotype-by-environment (GxE) interactions and their effect on individual performance under commercial rearing conditions.

Furthermore, with dense marker sets now available (Jones et al., 2017a,b; Guppy et al., 2018) it is also possible to rebuild deep pedigrees and examine the hidden cryptic relatedness between individuals (Jones et al., 2017b; Khatkar, 2017). Genomic relatedness matrixes (GRMs) can be generated which quantify the extent of the genome shared between individuals. This methodology characterizes the true relatedness between individuals more accurately than traditional pedigree or parentage assignment (Hayes et al., 2009). In particular, when applied to breeding programs with complex and cryptic family lines, GRMs are able to minimize inbreeding through careful selection and allocation of mate pairings (Nielsen et al., 2011; Jones et al., 2017b; Khatkar, 2017; Toro et al., 2017; Zenger et al., 2017). The transition to higher density marker panels (i.e., >3,000 SNPs) will increase the ability to definitively assign parentage of communally reared individuals, particularly as more polymorphic markers provide a higher capacity to determine relationships between inherently related individuals produced over multiple generations of breeding. GBS assays rather than low density solid state arrays may prove to be more versatile as they maintain an accessible genotyping cost, while providing required genotyping power (Robledo et al., 2017).

An alternative approach to increase marker density is genotype imputation. Imputation relies upon genotyping a small reference panel of individuals at high-density (e.g., 1,000 individuals) to generate robust allele frequency estimates. Once paired with genetic linkage information, the genotypes of high-density markers can be inferred within a second set of individuals (e.g., 10,000 individuals) genotyped with a low-density assay (Li et al., 2009). This approach, while statistically intensive, has proved effective for genotyping in breeding programs of terrestrial species (Pryce et al., 2014), and has been proposed to improve the cost effectiveness of large scale salmon genotyping (Tsai et al., 2017). Extensive modeling of the accuracy of imputed genotypes has been undertaken in terrestrial species (Hozé et al., 2013), but the accuracy of imputation is underpinned by factors including reference population size along with the size of chromosomal fragments (haplotype or haplotype blocks) that are co-inherited (García-Ruiz et al., 2015). Currently there is a poor understanding of length and rate of rearrangement (or recombination) of haplotypes within the penaeid genome (See section Linkage Mapping of Genetic Markers), impeding the current utilization of imputation for penaeid shrimp.

## Functional genomics

Examining the physiology, immunology, and genetics of penaeids at the transcriptional and translational level provides direct insight into the functional role of molecular mechanisms in the overall productivity of penaeid species, along with many other traits of biological and commercial interest (e.g., disease resistance, pigmentation, nutrition, reproduction). A number of methods are commonly employed to undertake this work, progressing from individual “gene by gene” characterisation (e.g., RT-qPCR, Sanger sequencing) through to profiling all transcriptionally active genomic elements simultaneously (i.e., RNA-seq). Proteomic analysis methods have traditionally focussed on characterisation and quantification of a small number of proteins (e.g., 2D gel separation), though more recent approaches can successfully characterize and quantify the complete constituency of the proteome (e.g., liquid chromatography coupled with mass spectrometry). As with other fields, functional genomics has been revolutionized by advances in next generation sequencing, mass spectrometry, and bioinformatics approaches.

### Expressed sequence tag sequencing and cDNA microarray development

Prior to the advent of high-throughput next-generation sequencing, and in the absence of a complete genomic sequence for penaeids, expressed sequence tag (EST) discovery and annotation was the focus of functional genomics studies for a significant period of time, with a substantial investment made in developing resources for research and commercial applications. Driven by the economic handicap that reproductive dysfunction and disease events have had upon the industry, these two areas have seen the majority of research effort applied.

In particular, EST studies have been conducted to identify potential genes of interest related to male (Leelatanawit et al., 2008, 2009) and female (Yamano and Unuma, 2006; Preechaphol et al., 2007, 2010) reproductive functions. Likewise, comparisons of EST libraries between challenged individuals (i.e., exposed to stress and pathogens), and non-challenged individuals, have allowed the identification of genes with putative roles in virally-induced immune response (Gross et al., 2001; Rojtinnakorn et al., 2002; Shen et al., 2004; Clavero-Salas et al., 2007; Dong and Xiang, 2007; Leu et al., 2007; Pongsomboon et al., 2008b; Maralit et al., 2014) and bacterial resistance (Bartlett et al., 2002; Supungul et al., 2002, 2004; de Lorgeril et al., 2005; Somboonwiwat et al., 2006). While these studies were effective in the initial discovery of a small number of penaeid genes (Table 3), alone they provide an incomplete view of the transcriptionally active genes in each biological situation investigated. Furthermore, additional complexity reduction steps [e.g., suppressive subtraction hybridisation (Leelatanawit et al., 2008; Preechaphol et al., 2010)] were required to avoid redundant repeat sequencing of highly expressed genes such as thrombospondin from ovary libraries, or 40S ribosomal proteins from gill libraries (Clavero-Salas et al., 2007; Preechaphol et al., 2007). Without these additional steps, studies such as those by Clavero-Salas et al. (2007) contained 69% redundancy in sequencing, limiting the novel gene sequences obtained.

**Table 3:** Summary of currently available functional genomics studies, including the generation of ESTs, microarrays, and transcriptome data.

**Technique**	Species^*^	**Factor examined**	**Tissues**	**Number of transcripts**	**Number of unigenes**	**Annotation Rate**	**References**
Expressed Sequence Tags (ESTs)	*L. vannamei*	WSSV	Gills	601 ESTs	–	87%	Clavero-Salas et al., 2007
	*L. vannamei*	Innate immune response	Hemolymph, eyestalk, hepatopancreas, lymphoid organ, nerve cord, gill	13,656 ESTs	–	38%	O'Leary et al., 2006
	*L. vannamei*	Antimicrobial peptides	Haemocytes	14 clones	–	100%	Bartlett et al., 2002
	*P. monodon*	♀ reproduction	Ovary	1,051 clones	559	–	Preechaphol et al., 2007
	*P. monodon*	♂ reproduction	Testis	896 clones	601	45.2%	Leelatanawit et al., 2009
	*P. monodon*	*Vibrio harveyi*	Haemocytes	1,062 ESTs	30^**^	10.8%	Supungul et al., 2004
	*P. monodon*	*Vibrio harveyi*	Haemocytes	267 clones	24	31%	Somboonwiwat et al., 2006
	*P. monodon*	*Vibrio harveyi*	Lymphoid organ	1,033 clones	22	25.2%	Pongsomboon et al., 2008b
	*P. monodon*	*Vibrio harveyi*	Haemocytes	615 ESTs	21^**^	44%	Supungul et al., 2002
	*P. monodon*	Gene discovery	Eyestalk, ovary, hepatopancreas, haematopoietic tissues, haemocyte, lymphoid organ	10,100 clones	4,845	48.8%	Tassanakajon et al., 2006
	*F. chinensis*	Innate immune response	Haemocytes	2,371 clones	482	49.6%	Dong and Xiang, 2007
	*F. chinensis*	Innate immune response	Cephalothorax	10,446 EST	3,120	44.0%	Xiang et al., 2008
	*L. setiferus*	Antimicrobial peptides/	Haemocytes	7 clones	–	100%	Bartlett et al., 2002
Subtractive Hybridisation	*P. monodon*	WSSV	Gills	9,597 ESTs	65	8.6%	Maralit et al., 2014
	*P. monodon*	♀ reproduction	Ovary	452	109	48.0%	Preechaphol et al., 2010
	*P. monodon*	♂ reproduction	Testis	365	71	45.5%	Leelatanawit et al., 2008
	*M. japonicus*	Sex and reproductive genes	Male and female gonads	296 transcripts	147, 36	7.3–43.8%	Callaghan et al., 2010
Microarray	*L. vannamei*	TSV, YHV	Hepatopancreas	2,469	–	–	Veloso et al., 2011
	*L. vannamei*	Immune response	Hemocytes, gill, hepatopancreas	7,021	2,469	36%	Robalino et al., 2007
	*P. monodon*	*Vibrio harveyi*, YHV, WSSV	Haemocytes	9,990 ESTs	420, 1,136, 1,954	15–25%	Pongsomboon et al., 2011
	*P. monodon*	YHV	Haemolymph	2,028	1,269	47% of the 105 DEG	Pongsomboon et al., 2008a
	*P. monodon*	WSSV	Hepatopancreas	47 ESTs, 37 cDNA	–	100%	Dhar et al., 2003
	*P. monodon*	WSSV	Haemocytes	1,026	30–135	52.4%	Wongpanya et al., 2007
	*P. monodon*	Stress	Hemocytes	3,853	–	17% of the 145 DEG	Vega et al., 2007
	*P. monodon*	♂ reproduction	Testis	4,803	2,702	41.9%	Wongsurawat et al., 2010
	*P. monodon*	♂ reproduction	Libraries from Tassanakajon et al. (2006)	10,536	5,568	70.6%	Leelatanawit et al., 2011
	*P. monodon*	♂ reproduction	Testis and vas deferens	6,072	–	42.6% of the 162 DGE	Leelatanawit et al., 2017
	*P. monodon*	♀ reproduction	Ovaries and testes	4,992	–	–	Karoonuthaisiri et al., 2009
	*M. japonicus*	♀ reproduction	Eyestalk	1,988	1,386	16.7%	Yamano and Unuma, 2006
	*M. japonicus*	YHV	Haemocytes	2,028	759	47% of 105 DEG	Pongsomboon et al., 2008a
	*F. chinensis*	WSSV	Cephalothorax	3,136 ESTs	–	–	Wang et al., 2006
	*F. chinensis*	WSSV	Hepatopancreas	–	59,137	75 genes^**^	Shi et al., 2016
Transcriptomics	*L. vannamei*	WSSV	Hemocytes	101,479 contigs	52,073	45.3%	Xue et al., 2013
	*L. vannamei*	WSSV	Hepatopancreas	–	14,583	73.24%	Chen et al., 2013
	*L. vannamei*	WSSV and growth	Hepatopancreas and muscle	63,105	14,124	33%	Santos et al., 2018
	*L. vannamei*	TSV	Hemolymph and hemocytes	61,937	–	20.0%	Sookruksawong et al., 2013
	*L. vannamei*	TSV	Hepatopancreas	–	15,004	69.5%	Zeng et al., 2013
	*L. vannamei*	Gonadal development	Testis and Ovaries	65,218	30,304	46.5%	Peng et al., 2015
	*L. vannamei*	Acute ammonia stress	Hepatopancreas	94,627	78,636	28.84%	Lu et al., 2016b
	*L. vannamei*	Osmoregulatory Stress	Hepatopancreas	26,034	38,237	25.2%	Chen et al., 2015
	*L. vannamei*	Osmoregulatory Stress	Gills	466,293	349,012	7.03%	Zhang et al., 2016
	*L. vannamei*	Nitrite	Hepatopancreas and hemocytes	92,821 contigs	42,336	55.6%	Guo et al., 2013
	*L. vannamei*	Embryo development	Whole larvae (20DAH)	162, 342 scaffolds	73,505	44.1%	Li et al., 2012
	*L. vannamei*	Larval Development	Embryo, Nauplius, zoea, mysis, post larvae	–	66,815	48.4%	Wei et al., 2014
	*L. vannamei*	Molting	Whole shrimp	–	93,756	16.6%	Gao et al., 2015
	*L. vannamei*	Feed efficiency	Muscle	–	72,120	28.63%	Dai et al., 2017
	*L. vannamei*	None specifically	muscle, hepatopancreas, gills, pleopods	110,474	87,307	24.4%	Ghaffari et al., 2014
	*P. monodon*	Growth	Heart, muscle, hepatopancreas and eyestalk	239,135 (raw)	69,089	17.8%	Nguyen et al., 2016
	*P. monodon*	Reproduction and development	Hepatopancreas and ovary	13,288 contigs	15,867	–	Rotllant et al., 2015
	*P. monodon*	Gene discovery	Eyestalk, stomach, female gonad, male gonad, gill, haemolymph, hepatopancreas, lymphoid organ, tail muscle, embryos, nauplii, zoea, and mysis, whole larvae (PL1, 4, 10, 15)	236,388	–	–	Huerlimann et al., 2018
	*M. japonicus*	Embryo development	Fertilized eggs, embryos and vegetal halves	46,781, 41,567	-	7.4–10%	Sellars et al., 2015
	*F. chinensis*	WSSV	Cephalothorax	-	46,676	46.28%	Li et al., 2013
	*F. merguiensis*	Color	Cuticle, muscle, androgenic gland, hepatopancreas, stomach, nervous system, eyestalk, male gonads, female gonads	5,990	-	59.8%	Ertl et al., 2013
	*F. merguiensis*	Reproduction and development	Hepatopancreas, stomach, eye stalk, nerve cord, male gonad, female gonad, androgenic gland region, muscle and cuticle	124,631	59,179 proteins	34.52%	Powell et al., 2015

* *L. vannamei (Litopenaeus vannamei), P. monodon (Penaeus monodon), M. japonicus (Marsupenaeus japonicus), F. chinensis (Fenneropenaeus chinensis), F. merguiensis (Fenneropenaeus merguiensis), L. setiferus (Litopenaeus setiferus)*;

** *Information on remaining transcripts/ESTs not reported*;

♂ *Male*,

♀ *Female*;

*WSSV, White spot syndrome virus; YHV, Yellow Head Virus; TSV, Taura Syndrome Virus*.

Through substantial investment in collaborative research programs, large scale multi-tissue and multi-life-stage studies have also been conducted with the aim of obtaining the complete transcriptional profile for penaeid species. A large number of novel ESTs have been identified in *P. monodon* (Tassanakajon et al., 2006), *L. vannamei* (O'Leary et al., 2006; Robalino et al., 2007), and *F. chinensis* (Dong and Xiang, 2007; Xiang et al., 2008), yet due to the small sequence length of each EST transcript (100–300 bp), the characterisation of transcriptional profile of these penaeids through classical sequence homology approaches has proved difficult. Many of these studies have been reviewed at length by Leu et al. (2011), with only an additional 20,318 EST tags deposited into the NCBI EST database since (was 196,248 in 2011, now 216,566 as of 8th May 2018; 709 for *P. indicus*, 5,609 for *L. vannamei*, 14,000 for *P. monodon*). While the number of ESTs is small in comparison to those generated for humans (>8 million) or Atlantic salmon (>500,000), valuable insight into genes involved in processes such as reproduction of penaeids has been obtained, with over 500 reproduction-related genes identified in *L. vannamei* and *P. monodon* (Leu et al., 2011).

Large sets of ESTs have been incorporated into a number of custom high throughput microarrays, which are able to provide relative expression profiles for 1,000's of genes simultaneously (Karoonuthaisiri et al., 2009; Wongsurawat et al., 2010; Leelatanawit et al., 2011). In particular, microarrays are now available for the study of reproductive function [ReproArray (Karoonuthaisiri et al., 2009; Wongsurawat et al., 2010), UniShrimpChip (Leelatanawit et al., 2011, 2017; Uawisetwathana et al., 2011)] and disease response within penaeids (Dhar et al., 2003; Wang et al., 2006; Wongpanya et al., 2007; Pongsomboon et al., 2008a, 2011; Veloso et al., 2011; Shi et al., 2016). While these studies have provided novel insight into functional genomics of penaeids (see review by Aoki et al., 2011), they provide limited novel sequence information and therefore limit the ability to differentiate between biologically significant isoforms or genetic variants. As such, high-throughput whole transcriptome sequencing approaches are instead becoming the preferred method to study the RNA profile of penaeids, due to their ability to simultaneously provide differential gene expression and sequence data.

### Transcriptome profiling with next generation sequencing

With the decreasing price of sequencing, improvements in library preparation, and bioinformatic techniques, it is possible to analyse complete cDNA libraries without the need for any complexity simplification steps. In particular, without the requirement to clone and individually sequence cDNA fragments (as is needed with EST sequencing), the ability to produce complete transcriptome profiles has increased dramatically (Li et al., 2012; Ghaffari et al., 2014; Santos et al., 2014; Huerlimann et al., 2018). It is now possible to isolate upwards of 50,000 genes, including rare variants in a single study (Table 3).

Like EST and microarray studies, the primary focus of transcriptomics studies has been immunology, disease resistance and reproductive biology. Screening experimentally challenged, or opportunistically sampled shrimp with infections, has resulted in the identification of a number of differentially expressed genes in *L. vannamei* (Chen et al., 2013; Sookruksawong et al., 2013; Xue et al., 2013; Zeng et al., 2013), *F. chinensis* (Li et al., 2013), *P. monodon* (Soonthornchai et al., 2016), and *F. merguiensis* (Powell et al., 2016). Through implementation of whole transcriptome sequencing, it has been possible to gain extensive insight into the host-virus interaction underpinning mass mortality events and has allowed researchers to develop potential methods to counter infectious disease events (Sellars et al., 2011; Li et al., 2013). Comparison between the immune response invoked by different viral pathogens has highlighted common innate immune pathways that may be useful for future selection of individuals based on broad spectrum immune response (Sookruksawong et al., 2013; Tassanakajon et al., 2013; Gao et al., 2015).

With the comparative ease of generating transcriptome profiles, a number of studies have not only isolated the molecular pathways responsible for tolerance to commonly observed environmental stressors such as nitrite (Guo et al., 2013) and ammonia (Lu et al., 2016a), but have also investigated their effect on productivity. For example, many genes and pathways linked to immune response (e.g., chitinase, peritrophin, thrombospondin, and peaeidin) and growth (linoleic acid metabolism) were identified by (Lu et al., 2016a) to be suppressed when investigating the effect of ammonia exposure in *L. vannamei*. Further research, linking environmental stressors (i.e., pH, salinity, and temperature) with studies on viral immunology, will provide a more complete understanding of the role that environmentally induced immune suppression plays in mass mortality disease events and decreased productivity.

Likewise, reproductive studies comparing male and female transcriptome profiles, along with various reproductive stages within sexes, has yielded novel insight into the molecular and genetic mechanisms underpinning maturation and sex determination of *L. vannamei* (Peng et al., 2015), *F. merguiensis* (Powell et al., 2015), and *P. monodon* (Rotllant et al., 2015; Goodall, 2017). As these studies are able to reconstruct complete (or near complete) RNA transcripts, it has been possible to analyse comprehensive gene pathways (Peng et al., 2015). Through the characterisation of functional gene regions it has been possible to deduce key gene to gene interactions and regulatory pathways for reproduction and maturation of male and female penaeids (Peng et al., 2015). For *P. monodon*, studies such as those undertaken by Rotllant et al. (2015) provide substantial opportunity to combat reproductive dysfunction in captive bred stocks (Arnold et al., 2013; Coman et al., 2013; Marsden et al., 2013). In particular, through differential gene expression analysis of whole-transcriptome data, genes related to fatty acid and steroid metabolism were found to have altered expression patterns when compared between wild sourced and domesticated stock (Rotllant et al., 2015). Similarly, exploration of the effects variable ovarian arachidonic acid content has on a number of key ovarian development pathways, revealed distinct differences between domesticated individuals (Goodall, 2017). Both studies provide a strong case for continued research into the informed improvement of reproductive maturation diets and may help the industry overcome commonly poor breeding performance (Rotllant et al., 2015; Goodall, 2017).

Interestingly, while functional genomics studies have also investigated the mechanisms involved in embryonic development (Li et al., 2012; Sellars et al., 2015) and molting (Gao et al., 2015), little whole transcriptome research has focused explicitly on muscle growth (Nguyen et al., 2016), to date being limited to confirmation of conserved growth genes, potentially overlooking important shrimp specific genes related to growth. Likewise, differential gene expression analysis undertaken by Dai et al. (2017) found expression of 383 genes relating to cell proliferation, growth, and energy and nutrient metabolism, showed distinct differences between selected lines with high and low feed efficiency, however only 57% (220) of these differentially expressed genes were annotated. Further research investment is required to gain an enhanced understanding of the functional genomic elements that dictate superior or inferior performance for both of these key economic traits.

As with earlier EST studies, functional annotation of data from transcriptome studies through classical sequence homology approaches has been difficult. Predominately, shrimp transcriptome studies are able to annotate between 20 and 50% of transcripts with Kyoto Encyclopedia Genes and Genomes (KEGG), Gene Ontology (GO), or NCBI databases (Table 3). As these database have inadequate penaeid sequence information, along with poor characterized sequence information from close relatives (Decapods, or Arthropoda), they hold limited utility in characterizing shrimp or crustacean specific transcripts which may be of high importance for traits such as disease resistance, reproduction, or growth (Huerlimann et al., 2018). Short-read based RNA sequencing (e.g., Illumina Hi-Seq) is high-throughput and relatively inexpensive, and as such has been the most widely applied method for recent penaeid transcriptomics studies. However, reconstructing full-length transcripts during transcriptome assembly is often problematic, with misassembles commonly observed, as well as poor representation of many alternatively spliced isoforms. In contrast, emerging long-read RNA sequencing methods (e.g., Pacific Bioscience Iso-Seq- or Nanopore Direct RNA sequencing) can sequence individual full-length RNA fragments and may provide increased insight into isoform diversity particular in non-model species (Kim et al., 2017; Xiaoxian et al., 2017). Further studies incorporating epigenomics methodologies such as whole genome bisulphite sequencing and DNA methylation analysis will also provide insight into regulation of RNA expression, however to date this research has been limited to development of a reduced cost methodology (He et al., 2015).

It is not possible to apply whole- transcriptome sequencing approaches on a commercially relevant scale (1000's of individuals). As such, a concerted effort needs to be applied to utilize existing data to develop commercially applicable transcriptomics tools for Penaeids. Unlike the extensive number available for livestock species there has been no commercially available tests developed from the transcriptomics research completed, despite the significant of number studies on penaeids to date (Table 3).

### Proteomics

Proteomics techniques have been applied across a range of aquaculture species (reviewed in Rodrigues et al., 2012), often with a focus on environmental toxicology. Within penaeid shrimp, proteomic analyses have been focused on understanding the effects of nutrition (Silvestre et al., 2010; Qiao et al., 2011), immunity (Robalino et al., 2009), viral infection (Chongsatja et al., 2007; Rattanarojpong et al., 2007; Wang et al., 2007; Bourchookarn et al., 2008; Robalino et al., 2009; Chai et al., 2010; Kulkarni et al., 2014; Li et al., 2014; Chen et al., 2016), bacterial infection (Wu et al., 2007; Somboonwiwat et al., 2010; Zhang J. et al., 2010; Chaikeeratisak et al., 2012), and environmental stress (Jiang et al., 2009; Fan et al., 2013, 2016; Xu C. et al., 2017). In much the same way as transcriptomics, these proteomic studies examine differentially expressed proteins from shrimp tissues under various pathogenic or environmental stressors compared with those under normal states. The most widely applied proteomics workflows to date have utilized a range of gel-based separations often over 2-dimensions (2D) that separates proteins by isoelectric point then by size. This is followed by image analysis, proteolytic digestion and identification of differentially expressed protein spots using various mass spectrometry (MS) or tandem mass spectrometry (MS/MS) techniques (Table 4). Although technically demanding, 2D gels are capable of identifying large numbers of unique protein spots, as well as determining post-translational modifications such as glycosylation and phosphorylation. However, quantifying differential changes in protein abundance in 2D requires specialized software, highly technical skills and establishment of appropriate intensity thresholds to prevent over or under estimations due to electrophoretic artifacts. This may allow the detection of several hundred protein spots, but vastly reduces the successful quantification or identification of proteins to between 10 and 50 proteins (Table 4). When successful, 2D gels have identified a small numbers of proteins that are likely to have functional significance under relevant culture or stress conditions, with some studies confirming as functional importance through complementary techniques (e.g., RNAi; Robalino et al., 2009).

**Table 4:** Summary of currently available penaeid proteomic studies.

**Technique**	**Species**	**Factor examined**	**Tissue**	**Proteins Identified**	**Differentially Expressed**	**Detailed technique**	**References**
2D LC-MS/MS	*P. monodon*	Antibiotics	Hemolymph	2394 spots 2 proteins	9	2D DIGE nanoLC-ESI-MS/MS	Silvestre et al., 2010
	*P. monodon*	*Vibrio harveyi*	Lymphoid organ	~1000 spots 20 proteins	27	2D gels nanoLC-ESI-MS/MS	Chaikeeratisak et al., 2012
	*P. monodon*	*Vibrio harveyi*	Hemocytes	nd spots 29 proteins	27	2D gels nanoLC-ESI-MS/MS	Somboonwiwat et al., 2010
	*P. monodon*	WSSV	Stomach	>200 spots 17 proteins	20	2D gels nanoLC-ESI-MS/MS	Kulkarni et al., 2014
	*P. monodon*	YHV	Lymphoid organ	370–420 spots 26 proteins	33	MALDI-TOF MS, nanoLC-ESI-MS/MS	Bourchookarn et al., 2008
	*L. vannamei*	WSSV	Gills	~500 spots 40 proteins	20	nanoLC-ESI-MS/MS	Chen et al., 2016
	*L. vannamei*	TSV	Hemocytes	320–400 spots 20 proteins	32	2D gels nanoLC-ESI-MS/MS	Chongsatja et al., 2007
	*L. vannamei*	WSSV	Gills	nd spots 37 proteins	37	Not described	Robalino et al., 2009
	*L. vannamei*	Vitamin C and Chinese herbs	Hemocytes Hepatopancreas	220–400 spots 10 proteins	29 28	2D gels MALDI-TOF/MS	Qiao et al., 2011
	*L. vannamei*	Cold stress	Hepatopancreas	913 spots 37 proteins	37	2D gels MALDI-TOF/TOF MS	Fan et al., 2016
	*L. vannamei*	Cold stress	Hemocytes	755 spots 30 proteins	30	2D gels MALDI-TOF/TOF MS	Fan et al., 2013
	*L. vannamei*	YHV	Gills	>200 spots 20 proteins	18	2D gels nanoLC-ESI-MS/MS	Rattanarojpong et al., 2007
	*L. vannamei*	WSSV	stomach	500 spots 53 proteins	27	2D gels nanoLC-ESI-MS/MS	Wang et al., 2007
	*F. chinensis*	Hypoxic stress	Hepatopancreas	620–640 spots 33 proteins	67	2D gels LC-ESI-MS/MS	Jiang et al., 2009
	*F. chinensis*	WSSV	Hemocytes	580 spots 33 proteins	45	2D gels MALDI-TOF MS/MS	Li et al., 2014
	*F. chinensis*	WSSV	Hepatopancreas	580–600 spots 81 proteins	81	2D gels MALDI-TOF/TOF MS	Chai et al., 2010
	*F. chinensis*	*Vibrio anguillarum*	Lymphoid organ	700 spots 95 proteins	17	2D gels nanoLC-ESI-MS/MS	Zhang J. et al., 2010
Shotgun Proteomics	*Penaeus monodon*	WSSV	Cuticular epithelium	429 proteins	12	iCAT or SCX MALDI-TOF/TOF-MS/MS	Wu et al., 2007
	*Litopenaeus vannamei*	Salinity	Hepatopancreas	533 proteins	84	iTRAQ isobaric labeling, SCX, nanoLC-MS/MS	Xu C. et al., 2017
	*Geocarcinus lateralis*	Eyestalk ablation	Y-organ	543 proteins	259	2D gels LC MALDI-TOF MS/MS	Lee and Mykles, 2006
	*Cancer borealis*	None	Pericardial organ Sinus glands (eyestalk) brain	142 proteins	–	MALDI FTMS; MALDI TOF MS/MS; nanoLC-ESI MS/MS	Ma et al., 2009

*ESI, electrospray ionization; FTMS, Fourier transform mass spectrometry; iCAT, isotope-coded affinity tags; LC, liquid chromatography; MALDI, matrix-assisted laser desorption/ionization; MS, mass spectrometry; MS/MS, tandem mass spectrometry; SCX, strong cation exchange; TOF, time of flight*.

Further methodological advances now allow analysis of complex mixtures of soluble proteins without the need for difficulties of running 2D gels and subsequently characterizing each protein spot. By coupling liquid chromatography (LC) with MS/MS in an approached termed shotgun proteomics, databases of known proteins can then be utilized to identify peptide mass fingerprints. Shotgun proteomics has yet to be applied widely to the study of penaeid shrimp, but holds potential to rapidly improve protein identification and functional characterisation. Of the only two examples to date in shrimp (Wu et al., 2007; Xu C. et al., 2017), the first applied two proteomic approaches, [sub-cellular fractionation and a cleavable isotope-coded affinity tag (iCAT)], each followed by peptide identification with matrix-assisted laser desorption/ionization tandem time of flight mass spectrometry (MALDI-TOF/TOF-MS/MS) to examine WSSV-infected shrimp. Once critical filtering and error rate calculations were performed, a total of 429 proteins were identified with a high degree of confidence. In addition, iCAT labeling was applied to assessing changes in protein abundance over a time series post-infection. The second used 8-plex isobaric tags for relative and absolute quantification (iTRAQ), followed by nano LC-MS/MS analysis to identify and quantify differently expressed proteins in the hepatopancreas of *L vannamei* exposed to low salinity (Xu C. et al., 2017). A total of 529 proteins were identified, of which 84 were statistically different between treatments. Both these studies represent an approximate 10-fold improvement in protein identifications over previous techniques (Table 4). Tissues that contain highly abundant proteins, such as hemocyanin contained within crustacean hemolymph, remain an obstacle to global scale identification of proteins using shotgun proteomics, but applications in other tissues or using protein labels await investigation.

The potential of shotgun proteomics has been demonstrated in other crustaceans, with studies of neuropeptide signaling pathways controlling molting in crabs successfully identified 543 proteins from the Y organ in the land crab *Geocarcinus lateralis* (Lee and Mykles, 2006) and 142 from *Cancer borealis* (Ma et al., 2009). These studies culminated in the proposition of a novel mechanism by which molt inhibiting hormone and nitric oxide synthase activates a signaling cascade of phosphoproteins that regulate molting and reproductive development (Lee and Mykles, 2006). A gel-free proteomic workflow using LC-ESI-MS/MS study revealed the identity of 62 proteins differentially expressed in hepatopancreas of prawn *Macrobachium rosenbergii* after exposure to the insecticide chlordecone (Lafontaine et al., 2017). Similar studies in penaeid shrimp could be employed to better understand the occurrence of reproductive dysfunction in domesticated shrimp stocks, in particular in *P. monodon*.

Recently, the combination of molecular weight cut-off during sample preparation (Wiśniewski et al., 2009) and unbiased MS analysis, called sequential window acquisition of all theoretical fragment ion spectra (SWATH), has provided a method to simultaneously detect and quantify thousands of proteins, without labeling or previous knowledge of the precursor peptide ions (Gillet et al., 2012; Chapman et al., 2014). As its name denotes, SWATH independently collects information based on time windows enabling detection of low-level ions rather than only selecting the most intense compounds in the sample as normally occurs with information dependent acquisition MS modes. Additionally, protein abundance quantified using SWATH has been shown to correlate with RNAseq transcriptomic data, as well as identify clinically relevant cancer biomarkers (Gao et al., 2017). SWATH information can also be combined with the more traditional multiple reaction monitoring mass spectrometry (MRM-MS) methods for protein quantification. MRM-MS is a double-level specificity tool to quantify specific targets in complex samples using existing knowledge of peptide *m/z* and fragment ion *m/z* transitions (Liebler and Zimmerman, 2013). The abundance of targets of interest is often assessed by three peptides and three transitions. Quantification of proteins employing MRM-MS has been successfully applied for the quantification of allergens in penaeids including *P. monodon* and other crustaceans (Korte et al., 2016) as well as for the detection of antibiotics in shrimp muscle (Tyagi et al., 2008).

As for most “omics” research, data accessibility through publicly available repositories is key to maintaining research quality and building upon past work. Examples of this include ProteomeXchange (http://www.proteomexchange.org) or the PRoteomics IDEntifications (PRIDE) database. These repositories contain curated MS spectral profiles that are standards compliant and facilitate peptide identification from existing proteomics data. High quality species-specific transcriptomes greatly improve peptide fragment and protein identification within proteomics workflows, even if that protein has no known function. While the penaeid research community may be dissuaded from publically releasing data due to the investment in data generation and perceived value, without concerted data sharing efforts, large proportions of data will become duplicated, while inevitably remaining poorly utilized due to incomplete characterisation of proteins and peptides.

For the purposes of this review, we have not included classical protein or gene function studies of small numbers of individual proteins through expression of recombinant proteins, pulldown assays, transient knockdown or western blotting. Complementary techniques such as targeted gene knockdown (RNAi and CRISPR), gene regulation (Methyl-seq, ChIPseq), protein-protein interactions (protein expression, pulldown assays), *in situ* hybridization, western blotting, cell culture and fluorescence microscopy can each aid in elucidating novel gene functions in shrimp. Overlaying and comparing results with those obtained in complementary metabolomics studies will also provide improved insight into functional response pathways to commercially relevant environmental stressors (Schock et al., 2013; Li et al., 2017) and diseases (Huynh et al., 2018).

## Dissecting and exploiting the genetic variation underlying phenotypes

One of the primary drivers behind integrating genomics research into food production industries is to gain an understanding of the relationship between genetic variation and phenotypes of commercially important traits (Abdelrahman et al., 2017). In particular, through quantitative trait locus (QTL) mapping, and genome-wide association studies (GWAS), it may be possible to identify the number, location and effect size of genetic elements (i.e., genes, loci, and regions) that are linked with the observed phenotypic variation of a trait (Mackay et al., 2009). At a fundamental level, these studies provide novel insight into the genetic basis and architecture of traits, and when findings are compared across species, they allow the study of evolutionary processes underpinning trait characteristics. Furthermore, on an applied level, identifying markers that are highly predictive for a superior or inferior phenotype is fundamental to improving the selection of elite individuals for breeding programs (Thorgaard et al., 2006). Recently, breeding programs for many agricultural species have utilized genome-wide estimated of performance (genomic breeding values), within the framework of genomic selection programs, to rapidly improve both simple and complex traits (Meuwissen et al., 2001, 2016). Similar efforts are to implement genomic selection underway for shrimp [*P. monodon* (Khatkar et al., 2017a), *L. vannamei* (Wang et al., 2016, 2017)], and promise to bring a substantial breeding improvements to the industry (Zenger et al., 2017).

### Quantitative trait loci mapping

As the availability of genomic resources for penaeid species has until recently been somewhat limited, the ability to undertake robust QTL studies has also been restricted. Early QTL studies examining penaeid production traits were limited in their power and ability to accurately isolate QTL effects (Alcivar-Warren et al., 2007; Du et al., 2010; Wang et al., 2012; Zhang et al., 2013). In particular, studies have been limited by incomplete genome coverage, along with the inclusion of insufficient individuals to ascertain precise QTL locations, or detect QTL of moderate or small effects. For instance, maps constructed and utilized by Wang et al. (2012) covered only 63% of the genome, and contained few markers (average of 5.1–5.4) per linkage group. At this resolution, it is possible to identify linkage groups that may contain QTL of interest, but isolating specific genes, or genomic regions, is generally not possible due to the large physical distance between markers (Alcivar-Warren et al., 2007; Andriantahina et al., 2013; Zhang et al., 2013). For instance Lyons et al. (2007), following on from research by Li Y. et al. (2006) and Li Z. et al. (2006), located a growth-related gene (EVOVL-like gene) through characterizing the region surrounding a growth-related QTL in *M. japonicus*. However, it remains unclear if this finding is indicative of the true genetic factor driving phenotypic variation, or if the association occurs through the co-localisation of a number of growth related genes in penaeids as suggested by Andriantahina et al. (2013).

Recently, a small number of QTL studies have been undertaken with vastly improved marker densities (3,959–4,626 SNPs), providing an improved ability to dissect the underlying genetic variation of traits. Robinson et al. (2014) utilized a SNP-based linkage map developed by Baranski et al. (2014) to investigate differences in survival time post-WSSV infection in *P. monodon*. Likewise, Yu et al. (2015) and Lu et al. (2016b) identified QTLs associated with growth in *L. vannamei*, and growth and high-temperature tolerance in *M. japonicus*. Unlike Robinson et al. (2014) both Yu et al. (2015) and Lu et al. (2016b) did not look to characterize the regions surrounding the identified QTLs, furthermore Lu et al. (2016b) did not compare findings with earlier growth QTLs observed by Li Y. et al. (2006). As such, while these studies provide the fundamental insight into the genetic architecture of these traits, they fall short of providing the opportunity to apply the findings to improve the selection of broodstock. Integration of dense linkage maps, transcriptome datasets, and genomic assemblies will facilitate the characterisation of QTLs and further extend the utility of the research findings and should be required for publication of future penaeid QTL studies.

### Genome-wide association studies

In the quantitative study of traits, the commonly excepted paradigm has largely shifted away from searching for the presence of a single gene or locus driving trait performance, to that where traits are more likely underpinned by tens to hundreds of genes, or regions of small effect, often dispersed across multiple chromosomes of the genome. Since it is possible to generate a high density of genome-wide markers, livestock, and crop species now predominately undertake a number of high resolution genome-wide association studies [GWAS; including regression models, linear mixed models, and a number of Bayesian mixed models (e.g., Bayes R, Bayesian Lasso, Bayes A)] in an effort to obtain a more accurate understanding of the number and distribution of trait associated loci along with their effect on commercially relevant traits.

To date, only two GWAS studies (Robinson et al., 2014; Khatkar et al., 2017b) have been published for penaeids. Both studies successfully identifying a number of loci significantly associated with sex confirming of observations of earlier QTL studies (Staelens et al., 2008) by concluding that sex in penaeids was likely a simple, genetically determined trait. Furthermore, Robinson et al. (2014) utilized three GWAS analysis approaches in tandem, to identify two markers associated with WSSV resistance (one marker with GRAMMAS and FASTA, one marker with QFAM). The limited identification of markers associated with this complex trait may stem from the limited samples size utilized (1,024 individuals) in the case the trait is highly polygenic, or as suggested previously by Hayes et al. (2010) there may be limited genetic variation underlying WSSV resistance of *P. monodon* (heritability < 0.001). While both markers were derived from RNA-Seq transcripts they were not further characterized in the study. Given the desire to selectively breed for disease resistance, further study of this trait, along with characterization of the markers associated should be completed to inform selective breeding programs.

In the second penaeid GWAS study, Khatkar et al. (2017b) found no significant associations of markers with the growth of *L. vannamei* after completing false-discovery-rate corrections for multiple tests. It is suggested by this study that no gene of large effect regulates growth, however, this finding contrasts those obtained earlier through QTL analysis by Yu et al. (2015) with the largest QTL found to explain 17.9% of phenotypic variation in *L. vannamei*. This may have resulted from the small number of phenotypic records used by Khatkar et al. (2017b), along with the use of genotypic information derived from pooled DNA samples. Furthermore additional individuals with genotype information and phenotypic records are required (~10,000) to undertake GWAS studies when including a large number of markers (5000–10,000), as each additional marker adds increasingly stringent multiple testing corrections (i.e., false-discovery-rate or FDR, Bonferroni corrections) that without sufficient detection power can eliminate otherwise potentially associated markers (Korte and Farlow, 2013). When investigating complex traits (those underpinned by multiple genomic regions of small effect) the expected effect size of each individual marker is small (each driving <1% of phenotypic variation) and can be overlooked by QTL and GWAS analysis methods, particularly when a small number of individuals (e.g., <5000–10,000) are utilized (Visscher et al., 2014). To date, no Bayesian association approaches have been used in GWAS, with linear mixed models (ASReml, FASTA, GRAMMAS) or linear regression (QFAM) employed instead. Use of Bayesian approaches may decrease bias in estimates of SNP effect size, by allowing SNP effects to be modeled from a mixture of normal distributions [i.e., small, medium, and large effect sizes (Goddard et al., 2016)]. These Bayesian approaches have been used in initial exploration of genomic selection models of penaeids, but did not explore or comment specifically on any associations found with traits (Wang et al., 2017).

### Use of genome-wide trait association information in penaeid breeding

While GWAS can identify loci underpinning the performance of traits, it is unlikely single or only a few markers can be utilized in isolation to select superior animals (e.g., Marker Assisted Selection, MAS), as a limited number of loci may not capture sufficient contribution of the true trait performance. Successful attempts to integrate MAS in aquaculture are limited, and are increasingly being overlooked in favor of alternative methods, and as such are not discussed in detail in this review. Like those of livestock species, panels containing 50–100s of markers [e.g., for feed efficiency in beef cattle (Abo-Ismail et al., 2018)] can be developed to allow low cost prediction of animal performance, but can often be poor at capturing sufficient trait variation to make accurate breeding decisions. Ultimately, it is expected as additional genomic resources become available and are integrated (i.e., linkage maps used for QTL studies are integrated into genomic assemblies), it will be possible to characterize trait associated genomic regions to the resolution of each underlying gene or possible causative mutations (Quantitative Trait Nucleotides, or QTN). However, due to the high commercial value of robust predictive markers for advanced penaeid breeding, it is likely that some research findings will be restricted from public scrutiny (Benzie, 2009). As an example of this, a patent (Vuylsteke et al., 2012) has been obtained for a sex-specific genetic marker identified by Staelens et al. (2008), due to the potential economic value of use within the production process of monosex populations of *P. monodon*. The publication of full datasets is often restricted due to the considerable investment required to generate large phenotypic and genotype datasets, along with an increasing recognition of the competitive value of genomic-based information generated under industry based partnerships.

In the long term, it is likely that selection for polygenic traits will instead be enhanced through genomic selection (GS), where genome wide information is used to ascertain genomic relationships among individuals, and combined with the effect size of each trait associated SNP markers to generate a genomic estimate breeding value (gEBV). Unlike the low density trait specific panels available for livestock, by utilizing genome-wide information GS allows recalculation and reconfiguration of the models underlying gEBVs, based on progressively updated genetic maps (e.g., linkage and LDU map). In addition, SNP effects are also continuously updated due to the observed LD decay between SNPs and trait locus across subsequent generations (Amaral et al., 2008). LD decay is expected to occur more rapidly given the relatively outbred status of penaeids in current and future breeding programs (Wang et al., 2016, 2017), when compared to most livestock species (e.g., cattle, sheep, horses, pigs, goats; Hall, 2016). Early modeling of genomic selection and prediction accuracy has been completed in *L. vannamei* (Wang et al., 2016, 2017), and indicates the ability to undertake genomic selection with a density of genome-wide markers (~3,200) currently available in commercial products (e.g., the Infinium ShrimpLD-24 v1.0 Bead Chip; Jones et al., 2017a). The effects of population stratification and marker number have been explored, however, prediction accuracy has only been estimated in internal subsets, rather than in subsequent novel generations. Mean reliability of the gEBVs (generated with BayesA, rrBLUP, and Bayesian LASSO) for body weight and body length, were 0.411 and 0.029 respectively (Wang et al., 2016), however, these values may decrease under empirical forward-prediction validation (Daetwyler et al., 2013). Additional samples [currently 200 individuals from 13 full-sib families (Wang et al., 2016)], as well as inclusion of half-sib families would provide more robust prediction capacity through improved estimates of SNP effects and division of maternal and paternal influences on trait performance. Further exploration of genomic selection breeding program designs (within- vs. between-family GS) and genotyping marker density should also be completed to ensure optimisation of genetic gain, in relation to cost of genotyping and inbreeding rates achieved (Lillehammer et al., 2013).

## Conclusions

The field of penaeid genomics has seen considerable research applied over the past 30 years. Recent advances in “omics” research has continued to push the industry toward broad-scale uptake of genomic resources, particularly toward the implementation of genomic selection, and has begun to provide the understanding to overcome a number of the impediments faced by industry. However, the resources available currently require further development to ensure wide-scale use is feasible, and that studies being undertaken provide the most informative results possible to aid industry development. With this in mind, suggestions for areas of research focus are;

- Incorporation of error-corrected long-read sequencing information and optical mapping to complete long standing genome assembly projects.
- Integration of resources (linkage maps, physical maps, annotated transcriptome, characterized proteome data, and genome sequence) to form a complete and highly informative dataset that can be provided in a genome browser
- Development and public provision of cost effective genotyping resources for use in commercial scale genomic selection programs, as well as quantitative traits studies (e.g., GWAS). Where possible consistent marker sets should be used to allow progressive improvement of datasets and comparison between studies.
- Development and release of high-density linkage maps (with minimal “binned” markers) and LDU maps to understand genome architecture, genome evolution, and inform genomic selection practices.
- Annotation of shrimp specific proteins and transcripts, as well as those conserved across taxa, to provide a complete understanding of the functional elements dictating shrimp performance. Cataloging of these should be maintained in public repositories to allow follow up studies (CRISPER or RNAi knockdown) to confirm role of identified targets of potential commercial importance.
- Undertaking of empirical forward-prediction genomic selection studies which incorporate traits of commercial importance (e.g., growth, disease resistance), to allow determination the most effective approach for the industry breeding programs to improve productivity and overcome current constraints (i.e., disease prevalence).

## Author contributions

All authors contributed significantly to the manuscript. The manuscript was prepared by JG. Manuscript structure was guided and first draft reviewed extensively by DBJ, DRJ, and KZ. Amendment and guidance was provided by HR, and RH. The section discussing proteomics was authored by NW. All authors reviewed the manuscript and approved its content.

### Conflict of interest statement

The authors declare that the research was conducted in the absence of any commercial or financial relationships that could be construed as a potential conflict of interest.
